# Definition, management, and training in impacted fetal head at cesarean birth: a national survey of maternity professionals

**DOI:** 10.1111/aogs.14600

**Published:** 2023-07-10

**Authors:** Katie Cornthwaite, Pauline Hewitt, Jan W. van der Scheer, Imogen A. F. Brown, Jenni Burt, Eliane Dufresne, Mary Dixon‐Woods, Tim Draycott, ABC Contributor Group, Thiscovery Authorship Group, Rachna Bahl

**Affiliations:** ^1^ Royal College of Obstetricians & Gynaecologists London UK; ^2^ Translational Health Sciences University of Bristol Bristol UK; ^3^ Royal College of Midwives London UK; ^4^ THIS Institute (The Healthcare Improvement Studies Institute), School of Clinical Medicine University of Cambridge Cambridge UK; ^5^ RAND Europe Cambridge UK; ^6^ North Bristol NHS Trust Bristol UK; ^7^ University Hospitals Bristol and Weston Bristol UK

**Keywords:** brain injury, cesarean birth, disimpaction, fetal pillow®, impacted fetal head, maternity, online, survey, training, vaginal push‐up

## Abstract

**Introduction:**

This study assessed views, understanding and current practices of maternity professionals in relation to impacted fetal head at cesarean birth, with the aim of informing a standardized definition, clinical management approaches and training.

**Material and methods:**

We conducted a survey consultation including the range of maternity professionals who attend emergency cesarean births in the UK. Thiscovery, an online research and development platform, was used to ask closed‐ended and free‐text questions. Simple descriptive analysis was undertaken for closed‐ended responses, and content analysis for categorization and counting of free‐text responses. Main outcome measures included the count and percentage of participants selecting predefined options on clinical definition, multi‐professional team approach, communication, clinical management and training.

**Results:**

In total, 419 professionals took part, including 144 midwives, 216 obstetricians and 59 other clinicians (eg anesthetists). We found high levels of agreement on the components of an impacted fetal head definition (79% of obstetricians) and the need for use of a multi‐professional approach to management (95% of all participants). Over 70% of obstetricians deemed nine techniques acceptable for management of impacted fetal head, but some obstetricians also considered potentially unsafe practices appropriate. Access to professional training in management of impacted fetal head was highly variable, with over 80% of midwives reporting no training in vaginal disimpaction.

**Conclusions:**

These findings demonstrate agreement on the components of a standardized definition for impacted fetal head, and a need and appetite for multi‐professional training. These findings can inform a program of work to improve care, including use of structured management algorithms and simulation‐based multi‐professional training.

## Graphical abstract

This survey offers the foundation for the first consensus‐based, standardized definition for impacted fetal head. It provides insights into the usefulness of non‐technical skills, demonstrates appetite for training across the multi‐professional team, and can inform a program of work to improve care.

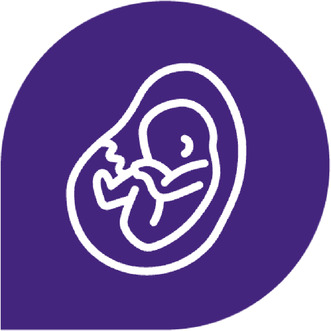

**Abbreviation**
IFH: impacted fetal head.

**Key message**

This survey offers the foundation for the first consensus‐based, standardized definition for impacted fetal head at cesarean birth. It provides insights into the usefulness of non‐technical skills for managing impacted fetal head, demonstrates appetite for training across the multi‐professional team, and can inform a program of work to improve care.

## 1 INTRODUCTION

Maternity professionals increasingly encounter impacted fetal head (IFH) at emergency cesarean births. Recent UK studies estimate that IFH may complicate 10% of cesarean births, or 1.5% of all births.^1^, ^2^ IFH is technically challenging and is associated with significant risks to mother and baby.^1^, ^3^, ^4^ Difficulty in disimpacting the fetal head can result in trauma to the uterus and bladder, postpartum hemorrhage and longer hospital stay.^1^, ^3^, ^4^ Babies are at increased risk of complications including skull fracture, intracranial hemorrhage, head and face trauma, low oxygen levels, admission to the neonatal intensive care unit and even death.^1^, ^3^, ^4^, ^5^ Reports of perinatal brain injury associated with IFH have risen, resulting in coronial enquiries, and increased litigation nationally and internationally.^5^, ^6^, ^7^, ^8^, ^9^ The NHS Resolution Early Notification Scheme identified IFH as a contributory factor in nearly 10% of potentially the most expensive UK maternity claims in 2018, making it twice as common as claims relating to cases of shoulder dystocia.^9^

A range of techniques can be employed to manage IFH,^3^, ^10^, ^11^, ^12^, ^13^, ^14^, ^15^ but there is no international consensus, beyond national committee opinions,^16^ on which are safest and most effective. Recent surveys of maternity professionals in the UK indicate a paucity of training, lack of confidence and under‐use of techniques that may be needed.^17^, ^18^ A standardized definition of IFH and an agreed approach to anticipation, communication and step‐wise management of IFH at cesarean birth have been lacking.^9^, ^18^ These gaps have likely contributed to variable practice and potentially harmful care in some circumstances.^18^ An exploration of the views of maternity professionals is an essential first step to improving care, training and research. We therefore undertook a survey of UK maternity professionals to assess their views, understanding and current practices in relation to IFH at cesarean birth, with the aim of informing the development of a standardized definition, clinical management approach and training.

## 2 MATERIAL AND METHODS

The survey was undertaken using Thiscovery (https://www.thiscovery.org/about), a secure online collaboration platform. It was targeted at qualified healthcare professionals currently providing care, or working in policy, research or other contexts relevant to maternity in the UK.

Participants were recruited from: Thiscovery subscribers who had previously signed up and consented to take part in activities relevant to maternity care; those who responded to targeted invitations from the Royal College of Midwives, Royal College of Obstetricians and Gynaecologists, Royal College of Anaesthetists, and other specialist networks such as the British Intrapartum Care Society, British Maternal & Fetal Medicine Society, the Obstetric Anaesthetists Association and Head of Midwifery/Consultant Midwife networks; and those who responded to invitations through social media channels.

The survey included questions about participants’ professional background, followed by combinations of closed‐ended and open‐ended questions that differed depending on professional background (Appendix S1). For example, all participants were asked about a multi‐professional approach and communication during IFH at cesarean birth. Midwives and obstetricians were asked about vaginal disimpaction (“vaginal push‐up”) and other disimpaction techniques, whereas obstetricians were asked about the criteria to define IFH by selecting from options informed by a previous survey of UK obstetric trainees.^18^

We undertook simple descriptive analysis of questions with closed‐ended responses. Count and percentages were calculated using R statistical software and Microsoft Excel for the total group and for professional subgroups (midwives, trainee/registrar/specialty obstetricians, consultant obstetricians).

Analysis of free‐text entries was based on qualitative content analysis, with a focus on manifest content of categories.^19^, ^20^, ^21^, ^22^ First, clinicians (KC, PH, TD, RB) and analysts (JWvdS, IAFB, MDW) generated six categories based on the topics and questions of the survey. Secondly, an analyst (IAFB) coded all free‐text entries to one of the six categories, verified by another analyst (JWvdS). If a participant's free‐text entry included more than one suggestion or comment, the entry was split, and coded as two or more separate responses. Thirdly, a clinician (PH) and two analysts (IAFB and JWvdS) generated subcategories “bottom‐up” (ie data‐driven) based on participant responses. The last step was an analyst (IAFB) counting the number of responses and the number of participants providing those responses, within categories and subcategories. Another analyst (JWvdS) verified these counts. Analyses were restricted to data of participants who completed all closed‐ended questions (complete‐case analysis excluding incomplete data of 38 participants).

### 2.1 Ethics statement

The study received ethical approval on October 25, 2021 from the University of Cambridge Psychology Research Ethics Committee (PRE.2021.089). All participants provided online consent before starting the survey.

## 3 RESULTS

A total of 419 participants consented to take part and completed all closed‐ended questions, including 144 midwives, 216 obstetricians and 59 other clinicians (Table 1), with participation spanning all regions of the UK (Table S1).

**TABLE 1:** Professional roles of survey participants (*N* = 419).

Professional role	*n* (%)
Midwives	144 (34)
Consultant obstetrician	145 (35)
Trainee/registrar/specialty obstetricians	71 (17)
Consultant anesthetists	30 (7)
Trainee/registrar/specialty anesthetists	6 (1)
Other (eg maternity support workers, academic staff)	23 (5)

### 3.1 Definition

When asked to select one or more components for a clinical definition, 79% (*n* = 171) of obstetricians preferred a definition including *“additional maneuvres and/or tocolysis to disimpact and deliver the fetal head”* (Table 2). About a third also preferred specifically referring to the delivering hand (ie difficulty or inability to get the usual delivering hand or either hand below the fetal head), with one‐quarter also preferring reference to *“deeply engaged in the pelvis”*. The 17 free‐text responses on this topic provided insight into why only about a third of participants selected a definition of IFH that incorporates the difficulty or inability of getting a hand below the fetal head to deliver it.

**TABLE 2:** Descriptions that should be part of a clinical definition of IFH at cesarean birth according to obstetricians (*n* = 216), with options informed by a previous survey of UK obstetric trainees.^18^

Description (one or more answers could be selected)	*n* (%)
“A cesarean birth that requires additional maneuvers and/or tocolysis to disimpact and deliver the fetal head”	171 (79)
“A cesarean birth where the obstetrician experiences **difficulty** getting their **usual delivering hand** below the fetal head to deliver it”	86 (40)
“A cesarean birth where the obstetrician is **unable** to get their **usual delivering hand** below the fetal head to deliver it”	74 (34)
“A cesarean birth where the obstetrician experiences **difficulty** getting **either hand** below the fetal head to deliver it”	73 (34)
“A cesarean birth where the obstetrician is **unable** to get **either hand** below the fetal head to deliver it”	67 (31)
“A cesarean birth where the fetal head is deeply engaged in the pelvis (at the level of or below the ischial spines)”	57 (26)

Abbreviation: IFH, impacted fetal head.

“I have found that in a recent case of impacted fetal head that I managed, I could get my hand below the head but it was wedged such that it could not be flexed or elevated.” [Trainee/registrar/specialty obstetrician]

One participant explained why ‘deeply engaged in the pelvis’ might not be appropriate for a definition of IFH, since *“some deeply engaged heads can be easily lifted from the pelvis”*. [Consultant obstetrician].

### 3.2 Multi‐professional approach

Virtually all participants (95%, *n* = 398) agreed or strongly agreed that management of IFH at cesarean birth requires a multi‐professional approach. Over 80% of participants indicated that midwives, trainee obstetricians, anesthetists, neonatologists, operating department practitioners and/or theater nurses would typically be present in theater for an emergency cesarean birth (Table S2). Only 38% (*n* = 157) indicated that consultant obstetricians would typically be present.

### 3.3 Communication

To communicate an IFH emergency with other team members, over half of participants preferred using the declaration *“This is an impacted fetal head”*, whereas few or no participants preferred *“The head is stuck”* or “*wedged”* (Table 3). Free‐text responses of 57 participants related to communicating an IFH, including 35 responses emphasizing the importance of good communication and awareness of the emergency among all team members both before and during management of IFH.

**TABLE 3:** Participants’ (*N* = 419) preferences for the phrase that is most appropriate to communicate an impacted fetal head (IFH) emergency to other team members, with options informed by a previous consultation of UK obstetricians and midwives.^44^

Phrase to communicate IFH to team	*n* (%)
“This is an impacted fetal head”	232 (55)
“I am having difficulty delivering the head”	103 (25)
“This is a deeply engaged fetal head”	44 (11)
“I am unable to deliver the head”	28 (7)
“The head is stuck”	5 (1)
“The head is wedged”	0 (0)
None, there is no need to declare this emergency	0 (0)
Other	7 (2)

“Everyone needs to understand that this is an emergency, and a protocol is being initiated, not just carrying on with individual tasks while the doctor performs a ‘difficult section’.” [Midwife]

Eleven responses stressed the importance of good communication with the woman and birth partner, including keeping them involved in discussions, thinking about the potential impact of clinical language used, and ensuring a structured form of communication.

“The fact of them and their birth partner being present and aware of what goes on in the theater needs to be formally taken into consideration when developing strategy recommendations including team communications. Hence a well formulated ‘script’ to use would be well received by teams.” [Trainee/registrar/specialty obstetrician]

### 3.4 Anticipation and management

Midwives and obstetricians largely agreed on level of suspicion of encountering IFH at cesarean birth in four given scenarios (Table 4). Midwives and obstetricians were least certain whether they should suspect IFH at cesarean birth with lack of progress in labor at 5 cm even with signs of obstruction in the form of significant caput and molding.

**TABLE 4:** How suspicious midwives and obstetricians (*n* = 360) were that an impacted fetal head (IFH) may be encountered at cesarean birth in various scenarios. Shaded boxes highlight the two most frequently selected levels of suspicion.

Scenario	Not at all suspicious	Slightly suspicious	Moderately suspicious	Very suspicious	Extremely suspicious
	*n* (%)	*n* (%)	*n* (%)	*n* (%)	*n* (%)
Low‐risk, multiparous woman at term. Delay in second stage of labor with an unsuccessful attempted assisted vaginal birth.	2 (1)	10 (3)	47 (13)	152 (42)	149 (41)
Induction of labor in low‐risk multiparous woman for estimated fetal weight >95th centile at 39 weeks. Cesarean section for deep transverse arrest at 8 cm cervical dilation.	4 (1)	23 (6)	84 (23)	129 (36)	120 (33)
Low‐risk, nulliparous woman at term. Augmented with oxytocin for slow progress at 5 cm cervical dilation. No progress since last vaginal examination, and evidence of significant caput and molding. Cesarean section for no progress in first stage of labor.	21 (6)	106 (29)	126 (35)	77 (21)	30 (8)
Nulliparous woman having induction of labor at 38 weeks for pre‐eclampsia. Cesarean section for presumed fetal compromise at 3 cm.	279 (78)	72 (20)	5 (1)	4 (1)	0 (0)

Over 70% of obstetricians indicated they found the following techniques and adjunctive measures acceptable (ie appropriate, safe and effective) for managing IFH prior to or at cesarean birth: change of operator, manual cephalic extraction (ie abdominal cephalic disimpaction in which the dominant or non‐dominant hand flexes and lifts the baby's head upwards into the maternal abdomen to deliver the head), tocolysis, operator changing hand, reverse breech extraction, Fetal Pillow®, head‐down tilt, and vaginal disimpaction (before and/or after incision) (Tables 5 and S3). Head‐down tilt was more frequently seen as acceptable by trainee/registrar/specialty obstetricians (94%) than by consultant obstetricians (79%) (Tables 5 and S3). Just under half of obstetricians (42%) viewed the Patwardhan method as an acceptable strategy (Tables 5 and S3). Some obstetricians considered potentially unsafe practices such as bladder filling (15%) or abdominal application of a single forceps blade (16%) or ventouse (10%), as acceptable (Tables 5 and S3).

**TABLE 5:** Techniques and adjunctive measures seen as acceptable (appropriate, safe and effective) in managing impacted fetal head at cesarean birth.

Techniques and adjunctive measures	Consultant obstetricians (*n* = 145)	Trainee, registrars and specialty obstetricians (*n* = 71)
	*n* (%)	*n* (%)
Change of operator (different clinician)	139 (96)	68 (99)
Manual cephalic extraction using usual delivering hand	139 (96)	69 (97)
Tocolysis (GTN/terbutaline/salbutamol)	129 (89)	67 (94)
Operator changing hand to perform manual cephalic extraction	127 (88)	70 (99)
Reverse breech extraction	122 (84)	70 (99)
Fetal Pillow® (fetal head elevating device)	118 (81)	54 (76)
Head‐down tilt	115 (79)	67 (94)
Vaginal push‐up (after incision)	109 (75)	55 (78)
Vaginal push‐up (pre‐incision)	101 (70)	55 (78)
Patwardhan method (shoulders first)	61 (42)	29 (41)
Bladder filling	19 (13)	13 (18)
Single forceps blade	21 (15)	13 (18)
Ventouse	15 (10)	7 (10)
Tydeman tube	14 (10)	2 (3)
C‐snorkel	8 (6)	2 (3)

Abbreviations: GTN, glyceryl trinitrate.

Free‐text responses by 56 participants suggested mixed views on the use of tocolysis for managing IFH at cesarean birth, with some associating it with risk of complications such as postpartum hemorrhage. Use of Fetal Pillow® was mentioned, largely positively, by 71 participants, but there were also calls for improved clarity and better evidence.

“It would be good to know if it [Fetal Pillow®] is effective or not, as lots of pressure to introduce it might help. Many Trusts have introduced it following incidents, and the Trainees like it.” [Consultant Obstetrician]

Free‐text responses by 55 participants highlighted the need to consider the position of the table in relation to the operator. Suggestions included lowering the table, having a step for the operator or using head‐down tilt.

About three‐quarters of obstetricians considered vaginal disimpaction acceptable prior to cesarean birth or for management of IFH during cesarean birth (pre‐incision: 72%; post‐incision: 76%) (Table 5), but it appeared to be less favored by midwives (pre‐incision: 25%, *n* = 36; post‐incision: 57%, *n* = 82) (Table S3). Most midwives and obstetricians (88%, *n* = 317) reported that it would be helpful to know the position of the fetal head before undertaking vaginal disimpaction. An important minority of obstetricians (20%, *n* = 44) and over a third of midwives (38%, *n* = 55) did not report using their whole hand to perform vaginal disimpaction. A relatively large number of midwives reported using the same (two‐finger) technique as for standard vaginal examination (22%, *n* = 32), without re‐positioning the woman's legs from a supine position (27%, *n* = 39) (Table S4). Eleven participants commented on the importance of vaginal disimpaction prior to cesarean birth if IFH is anticipated. Twenty‐two participants referred to reasons why maternity professionals may not do vaginal disimpaction, mostly citing a perceived risk or fear of fetal skull fractures.

“Push up [vaginal disimpaction] was performed both pre incision and post and the baby was later found to have a skull fracture. They advised that push up was therefore no longer allowed and asked for staff to use a fetal pillow instead.” [Trainee/registrar/specialty obstetrician]

### 3.5 Training

Most midwives and obstetricians (*n* = 309, 85%) considered training in vaginal disimpaction essential. Yet most midwives (*n* = 117, 81%) and over half of obstetricians (*n* = 92, 57%) indicated they had not received training for performing vaginal disimpaction. The most frequently preferred training method across all participants was hands‐on training in simulation (91%, *n* = 380). Others also preferred animated video illustrating disimpaction techniques (73%, *n* = 306), small‐group teaching (55%, *n* = 229), hands‐on training in real‐life (44%, *n* = 184), augmented reality (30%, *n* = 124) or lecture‐based teaching (19%, *n* = 78). Free‐text responses from 159 participants reflected the importance of training, and a need and appetite for it.

“I believe teaching to understand exactly what the mechanism is for the head impaction and then understanding the direction and angle of the use of your hand to release the head abdominally is essential. […] It needs to be demystified by being able to see it demonstrated in the same way that this was, and is now routinely done, for demonstrating the effective management of shoulder dystocia.” [Consultant Obstetrician]

### 3.6 Improving care

Free‐text responses from 100 participants included suggestions on improving care in relation to IFH at cesarean birth. Most frequently mentioned were briefing and debriefing before and after the emergency (33 participants) and the importance of robust plans for escalation and calling for help (26 participants).

## 4 DISCUSSION

In this survey of 419 maternity professionals involved in managing IFH, we found high levels of agreement on the components of an IFH definition (79% of obstetricians) and use of a multi‐professional approach to IFH management (95% of all participants). There was strong support for consistent use of shared language when communicating the emergency within the team. Acceptability of methods for management of IFH was particularly high for change of operator, abdominal cephalic disimpaction, tocolysis, operator changing hand, reverse breech extraction, Fetal Pillow®, head‐down tilt and vaginal disimpaction. Some obstetricians considered a number of potentially unsafe practices as appropriate. Availability of training for IFH management was variable, with over 80% of midwives reporting no training in vaginal disimpaction.

IFH at cesarean birth is a challenging, high‐risk obstetric emergency with a relatively high incidence,^1^, ^2^, ^23^ yet it has escaped consensual definition to date. Our survey has identified agreement on relevant defining components, based on selection of one or more previously identified definitional components,^18^ supplemented by free‐text responses. This leads us to suggest a standard pragmatic definition for potential use in practice, training, audit and research:

*A cesarean birth where the obstetrician is unable to deliver the fetal head with their usual delivering hand, and additional maneuvres and/or tocolysis are required to disimpact and deliver the fetal head*.

We also found appetite for consistent use of language during clinical scenarios involving IFH. This approach is supported by evidence from other obstetric emergencies, such as shoulder dystocia, showing that clearly and calmly declaring the emergency using unambiguous terminology facilitates teamworking, communication and management.^24^ Our survey suggests a preference for the declaration *“This is an impacted fetal head”*. Very few or no participants chose more vernacular phrases (*“The head is wedged”, “The head is stuck”)*, perhaps because these might be more alarming for the person in labor. Free‐text responses also emphasized the need to consider communication with the woman and birth partner as part of the IFH management approach.

Also important is awareness of the likelihood of IFH and the circumstances under which it can occur.^9^ Most midwives and obstetricians correctly classified scenarios with increased risk (unsuccessful assisted vaginal birth and deep transverse arrest) but there appeared to be less awareness that IFH is not confined to cesarean birth at full cervical dilation.^1^, ^2^ Given emerging evidence that IFH may be as common in cesarean birth performed prior to full cervical dilation,^1^, ^2^ maternity professionals should be prepared to encounter an IFH at all emergency cesarean births, and be trained accordingly.

The right skills and interventions are needed to manage this emergency,^18^ yet our survey identified variability in the range of expertise typically available in theaters, and support (albeit limited) for some potentially unsafe practices. Almost 20% of trainee/registrar/specialty obstetricians considered bladder filling acceptable, apparently unaware that – unlike situations like cord prolapse where the fetal head is high – bladder filling is unlikely to be effective where the head is impacted low in the pelvis, and could increase the risk of bladder injury during peritoneal entry at cesarean birth.^25^ Moreover, over 10% of consultant obstetricians deemed abdominal application of a single forceps blade and/or ventouse acceptable – yet these interventions may increase the risk of injury to women and babies, and should not be used in the absence of supporting evidence.^5^, ^26^, ^27^

For vaginal disimpaction, our survey indicated that over 20% of midwives and over 10% of obstetricians may use the same (two‐finger) technique as standard for vaginal examination, and not re‐position the woman's legs at all. A coroner's report and clinical descriptions of vaginal disimpaction indicate it is imperative that a whole hand is used to cup the baby's head in order to flex and elevate it, while avoiding focal pressure points on the fetal skull.^8^, ^10^, ^11^, ^28^ This is easier to perform if it is possible to provide adequate vaginal access through flexion and abduction of both of the woman's legs.^10^, ^11^, ^28^

Safe and effective vaginal disimpaction depends on skill, knowledge and manual dexterity, but does not appear to be consistently or routinely taught,^18^ with more than 80% of midwives reporting lack of training. There was evidence of uncertainty and anxiety among midwives about vaginal disimpaction, possibly related to reports attributing neonatal injury to vaginal disimpaction.^8^, ^29^ Consultants (who are more likely to be expert) are not always present or available during an IFH. Moreover, relying on training of obstetricians alone is likely suboptimal given the body of evidence on multi‐professional training.^30^, ^31^, ^32^, ^33^

Implementation of nationally standardized multi‐professional training could help improve safety, increase professional confidence, and enhance experiences of those in labor, their partners, and professional teams. Interventions to improve management and training must, however, be designed in the knowledge that IFH is unpredictable, and that the techniques are difficult to learn through direct experience.^18^ It is also critical that training emphasizes both the technical and non‐technical skills, including those relating to anticipation and preparation, communication and shared understanding, teamwork and behavior, and decision‐making,^24^, ^34^, ^35^ with specific care to communicating with service users during emergency healthcare provision.^36^, ^37^, ^38^, ^39^, ^40^ Structured management algorithms may be especially helpful in facilitating transfer of knowledge from experts to novices, and can be used in both real‐life and simulation training.^41^, ^42^

This is the largest survey to explore the management of IFH among the range of professionals involved in emergency cesarean births in UK practice, accessing a breadth of views so far lacking in the literature. It offers the foundation for the first consensus‐based, standardized definition for IFH. It expands previous work^17^, ^18^ on clinical management techniques by providing insights into the usefulness of non‐technical skills, and demonstrates appetite for technical and non‐technical training across the multi‐professional team. Other strengths of the study include a multi‐disciplinary approach used in devising the survey, the inclusive methods used for conducting the study, and independent analysis of the data by health services analysts to minimize clinical bias.

The sample represents a relatively small proportion of the population of maternity professionals in the UK. Notwithstanding, the wide participation of various professionals across the UK and alignment of our findings with previous reports offers confidence that similar results would be found in a larger sample.^17^, ^18^, ^24^ Our use of complete‐case analysis may have introduced some biased estimates if there were differential responses to particular questionnaire items.^43^ Finally, the sample size precluded statistical comparison of subgroups, limiting interpretation of differences between professional groups.

## 5 CONCLUSION

There is high agreement among UK maternity professionals on components of a definition of IFH at cesarean birth. Their views substantiate the importance of using safe and effective techniques in parallel with non‐technical skills for managing IFH, and the need for standardized management approaches and high‐quality training. These findings can inform a program of work to improve care, including use of structured management algorithms and simulation‐based multi‐professional training.

### Authorship groups

ABC Contributor Group: Wendy Randall, Chloe Hughes, Caroline Walker, Sarah Blackwell, Louise Dewick, Lisa Hinton, Akbar Ansari, Bethan Everson, Giulia Maistrello, Emily Hotton.

Thiscovery Authorship Group: Ruth Cousens, Jordan Moxey, Luke Steer, Andy Paterson, André Sartori.

## AUTHOR CONTRIBUTIONS

KC contributed to conceptualization, formal analysis, investigation, methodology, project administration and writing – original draft preparation. PH contributed to formal analysis, investigation, methodology and project administration. JWvdS contributed to data curation, formal analysis, investigation, methodology, project administration and writing – original draft preparation. IAFB contributed to data curation, formal analysis and project administration. JB contributed to conceptualization and methodology. ED contributed to data curation, formal analysis and software. MDW contributed to conceptualization, formal analysis, funding acquisition, methodology, resources, supervision and writing – original draft. TD contributed to conceptualization, funding acquisition, methodology and supervision. RB contributed to conceptualization, formal analysis, funding acquisition, investigation, methodology, project administration, supervision and writing – original draft. All authors contributed to writing – review and editing, and read and approved the final article.

## FUNDING INFORMATION

The Avoiding Brain Injury in Childbirth program (Department of Health and Social Care, UK) supported this work. THIS Institute is funded by the Health Foundation, Grant/Award Number: RHZF/001–RG88620. The Health Foundation is an independent charity committed to bringing about better health and healthcare for people in the UK. Mary Dixon‐Woods is an NIHR Senior Investigator (NF‐SI‐0617‐10 026).

## CONFLICT OF INTEREST STATEMENT

The authors have stated explicitly that there are no conflicts of interest in connection with this article.

## Supporting information


Appendix S1.

Table S1.
Table S2.
Table S3.
Table S4.
