# Supplementary material for: Definition, management, and training in impacted fetal head at cesarean birth: a national survey of maternity professionals
Source: Acta Obstet Gynecol Scand. 2023 Jul 10;102(9):1219–26. doi: 10.1111/aogs.14600 (PMC10407013; doi:10.1111/aogs.14600)
Supplement: Supplementary file 1 — Appendix S1. [file AOGS-102-1219-s002.pdf]

Supplementary Material 1. Survey questionnaire

The survey was undertaken using Thiscovery (https://www.thiscovery.org/about), an online research and development platform.

About you and your unit (all participants)

Before we start, can we ask some information about your role?

- What is your professional role?
  - Midwife band 5 – 7
  - Midwife band 8 – 9
  - Consultant Obstetrician
  - Trainee, SAS and Trust Doctor Obstetrician*
  - Consultant Anaesthetist
  - Trainee, SAS and Trust Doctor Anaesthetist*
  - MSW (Maternity Support Worker)
  - HCA (Health Care Assistant)
  - Student Midwife
  - I am not listed above, please specify: [free-text box]

    *Choose one of these options if you are at any stage of training, or are a SAS (Staff Grade, Associate Specialist, Specialty Doctor, Specialist or any other closed grade) doctor, or a Trust / Locally-employed doctor.

- Do you hold any clinical leadership roles?
  - Yes
  - No

- What region in the UK do you work?
  - East of England
  - London
  - Midlands
  - North East and Yorkshire
  - North West
  - Northern Ireland
  - Scotland
  - South East
  - South West
  - Wales

- What type of maternity unit or setting do you currently work in? If you work in more than one setting, please select all options that apply.
    - Obstetric unit
    - Alongside midwifery unit (a midwifery-led unit or birth centre situated in the same hospital or on the same site as an obstetric unit)
    - Freestanding midwifery unit (a midwifery-led unit or birth centre not situated in a hospital or site with an obstetric unit)
    - Community
    - Other, please specify: [free text]

- It would be really helpful if you could provide the name of the trust/s you are employed by, if applicable:

- "In the coming months, we'll be doing more work on the Avoiding Brain Injury in Childbirth (ABC) project. Are you happy for us to use information you have given about your role to contact you about this future work or other projects that may be relevant to you?
    - Yes
    - No

Background information presented to participants prior to starting the questions on IFH at caesarean section

Maternity staff are increasingly encountering impacted fetal head (IFH) at the time of caesarean section.

Complications associated with IFH have risen sharply in recent years. But currently there is no national guidance, consensus for best practice, or evidence-based training.

We would like to understand your views on how best to manage and train for this critical obstetric emergency.

We will now ask you **some questions** about your views on approaches to management and multi-disciplinary training for IFH at caesarean section. Your responses will help us explore how best to refine the outline approach, and will inform future training.

The activity should take no longer than **10 to 15 minutes** to complete.

Questions for all participants:

Question 1. To what extent do you agree with this statement:

‘The management of IFH at caesarean section requires a multi-disciplinary approach’

Strongly agree	Agree	Neither agree nor disagree	Disagree	Strongly disagree

Question 2: In your maternity setting, which healthcare professionals would typically be present in theatre for an emergency caesarean section?

[Please select all that apply]

- Midwife band 5 – 7
- Midwife band 8 – 9
- Consultant Obstetrician
- Trainee, SAS and Trust Doctor Obstetrician *
- Consultant Anaesthetist with regular sessions in obstetrics
- Consultant Anaesthetist without regular sessions in obstetrics
- Trainee, SAS and Trust Doctor Anaesthetist* on obstetric rotation
- Trainee, SAS and Trust Doctor Anaesthetist* not on obstetric rotation
- Operating department practitioner
- Theatre nurse
- Consultant neonatologist
- Trainee, SAS and Trust Doctor* neonatologist
- Advanced neonatal nurse practitioner
- MSW (Maternity Support Worker)
- HCA (Health Care Assistant)
- Other, please specify: [free text box]

*These options include doctors at any stage of training, or SAS (Staff Grade, Associate Specialist, Specialty Doctor, Specialist or any other closed grade) doctors, or Trust / Locally-employed doctors.

Question 3: How would you like training for IFH at caesarean section to be delivered?

[Please select all that apply]

- Hands-on training in real-life
- Hands-on training in simulation
- Small group teaching
- Lecture based teaching
- Animated video showing how to perform disimpaction techniques
- Augmented reality supported training
- Other – please specify: [free text box]

Question 4: Which of the following phrases do you think is most appropriate to communicate to the team that there is an IFH?

“This is an impacted fetal head”
“This is a deeply engaged fetal head”
“The head is stuck”
“The head is wedged”
“I am unable to deliver the head”
“I’m having difficulty delivering the head”
None – there is no need to declare this emergency
Other – please specify: [free text box]

[All participants except Midwives band 5 – 7, Midwives band 8 – 9, Consultant Obstetricians and Trainee, SAS, trust doctor obstetricians are directed to the free-text box questions at the end **(i.e. skip to Questions 10 and 11)**

Questions for obstetricians and midwives only

Question 5: How suspicious would you be that an IFH may be encountered at caesarean section in the following scenarios?

- Low-risk, multiparous woman at term. Delay in second-stage of labour with an unsuccessful attempted assisted vaginal birth.

Not at all suspicious	Slightly suspicious	Moderately suspicious	Very suspicious	Extremely suspicious

- Low-risk, nulliparous woman at term. Augmented with oxytocin for slow progress at 5cm cervical dilatation. No progress since last vaginal examination, and evidence of significant caput and moulding. Caesarean section for no progress in first stage of labour.

Not at all suspicious	Slightly suspicious	Moderately suspicious	Very suspicious	Extremely suspicious

- Nulliparous woman having induction of labour at 38 weeks for pre-eclampsia. Caesarean section for presumed fetal compromise at 3cm.

Not at all suspicious	Slightly suspicious	Moderately suspicious	Very suspicious	Extremely suspicious

- Induction of labour in low risk multiparous woman for estimated fetal weight > 95th centile at 39 weeks. Caesarean section for deep transverse arrest at 8cm cervical dilatation.

Not at all suspicious	Slightly suspicious	Moderately suspicious	Very suspicious	Extremely suspicious

Question 6: Which of the following methods do you see as acceptable (safe and effective) or unacceptable (unsafe and/or ineffective) in preventing or managing IFH at caesarean section?

What do we mean by acceptable? Please click here. [pop-up box]

"Several strategies are described and used to prevent and manage IFH at caesarean section. However, there is no consensus regarding which is the safest or most effective, particularly in relation to neonatal outcomes. We would like to know your opinion on which methods you consider to be appropriate, safe and effective (acceptable), and which you consider unsafe and / or ineffective (unacceptable)."

Manual cephalic extraction using usual delivering hand
- Acceptable
- Unacceptable
- I don't know

Operator changing hand to perform manual cephalic extraction
- Acceptable
- Unacceptable
- I don't know

Change of operator (different clinician)
- Acceptable
- Unacceptable
- I don't know

Vaginal push-up (pre-incision)
- Acceptable
- Unacceptable
- I don't know

Vaginal push-up (after incision)
- Acceptable
- Unacceptable
- I don't know

Reverse breech extraction
- Acceptable
- Unacceptable
- I don't know

Fetal pillow (fetal head elevating device)
- Acceptable
- Unacceptable
- I don't know

Patwardhan method (shoulders first)

- Acceptable
- Unacceptable
- I don’t know

Tocolysis (GTN / Terbutaline / Salbulatmol)

- Acceptable
- Unacceptable
- I don’t know

Single forceps blade

- Acceptable
- Unacceptable
- I don’t know

Ventouse

- Acceptable
- Unacceptable
- I don’t know

Head down tilt

- Acceptable
- Unacceptable
- I don’t know

Tydeman tube

- Acceptable
- Unacceptable
- I don’t know

C-snorkel

- Acceptable
- Unacceptable
- I don’t know

Bladder filling

- Acceptable
- Unacceptable
- I don’t know

Other acceptable strategy- please specify: [free text box]

Question 7: In my current practice, we perform a vaginal push-up in the following way:

[Please select all that apply]

Technique

Using the same technique as for standard vaginal examination
Using a whole hand to disimpact the fetal head

Positioning

Flex and abduct **both** of the woman's legs **prior to commencing the caesarean section**, if there is an increased risk of IFH
Flex and abduct **both** of the woman's legs **after an IFH is diagnosed** and when the decision is made to perform a vaginal push-up
Flex and abduct **one** of the woman's legs to achieve access to perform the vaginal push-up
Perform the manoeuvre without re-positioning the woman's legs from a supine position

Other

Other- please specify: [free text box]
None of the above [unique answer option]

Question 8: We would like to know a little more about your views on training for the vaginal push up method.

- Do you consider it helpful to know the position of the fetal head before undertaking a vaginal push up?
    - Yes
    - No
    - I don't know

- Do you consider it essential to have received training in how to perform a vaginal push-up in the event of this obstetric emergency?
    - Yes
    - No
    - I don't know

- Have you received training for how to perform a vaginal push up?
    - Yes
    - No
    - I don't know

[All participants except Consultant Obstetricians and Trainee, SAS, trust doctor obstetricians are directed to the free-text box questions at the end **(i.e. skip to Questions 10 and 11)**

Question for <u>obstetricians only</u>

Question 9: Which of the following descriptions should be part of a clinical definition of IFH at caesarean section? ***Select one or more that apply***

0 ‘A caesarean birth where the fetal head is **deeply engaged** in the pelvis (at the level of or below the ischial spines)’
0 ‘A caesarean birth where the obstetrician is **unable** to get their **usual delivering hand** below the fetal head to deliver it’
0 ‘A caesarean birth where the obstetrician experiences **difficulty** getting their **usual delivering hand** below the fetal head to deliver it’
0 ‘A caesarean birth where the obstetrician is **unable** to get **either hand** below the fetal head to deliver it’
0 ‘A caesarean birth where the obstetrician experiences **difficulty** getting **either hand** below the fetal head to deliver it’
0 ‘A caesarean birth that requires **additional manoeuvres** and/or tocolysis to disimpact and deliver the fetal head’
0 None of the above [unique answer option]
0 The following (additional) description: ... [free text]

Please use the box below to provide any additional comments on the definition of IFH

[Free text box]

Final questions and thank you for <u>all</u> participants

Question 10: We would like to know what interventions or actions you are aware of that enable staff to improve safety in relation to the management of IFH at caesarean section. Please note down anything that comes to mind below.

> ***Why are we asking this? Please click here. [Pop-up box]***
>
> *"We know there are lots of examples of good practice to undertake reflective learning and improve safety culture. We would like to hear about anything you are aware of that enables staff to reflect on and improve care. We will collate this at a national level, to inform the design of a lessons learnt approach to support the reduction of brain injury in childbirth."*

[Free text box]

Question 11. Is there anything else you would like to share about your views on approaches to the management of, and multi-disciplinary training for, IFH at caesarean section?

[Free text box]

Thank you

Thank you for participating in this survey: your time and input are very much appreciated. Your responses will be confidential.

We will collate the findings that you and other maternity staff have provided in this survey and plan next steps for the collaborative approach to impacted fetal head at caesarean section.
