# Supplementary material for: Definition, management, and training in impacted fetal head at cesarean birth: a national survey of maternity professionals
Source: Acta Obstet Gynecol Scand. 2023 Jul 10;102(9):1219–26. doi: 10.1111/aogs.14600 (PMC10407013; doi:10.1111/aogs.14600)
Supplement: Supplementary file 2 — Table S1. Table S2. Table S3. Table S4. [file AOGS-102-1219-s001.pdf]

Supplementary Tables S1 to S4

Table S1. Clinical practice region of survey participants (N=419)

Region	n	(%)
East of England	34	(8)
London	35	(8)
Midlands	56	(14)
North East and Yorkshire	46	(11)
North West	41	(10)
Northern Ireland	13	(3)
Scotland	20	(5)
South East	55	(13)
South West	76	(18)
Wales	21	(5)
Did not report	22	(5)

Table S2. Healthcare professionals reported as typically present in theatre for an emergency caesarean birth (n=419)

**Healthcare professionals that could be typically present in theatre for an emergency caesarean section** (one or more answers possible)	**n**	**(%)**
Trainee obstetrician (including registrars and SAS doctors)	402	(96)
Midwife band 5-7	403	(96)
Trainee anaesthetist on obstetric rotation (including registrars and SAS doctors)	375	(90)
Operating department practitioner	375	(90)
Trainee neonatologists (including registrars and SAS doctors)	353	(84)
Theatre nurse	349	(83)
Advanced neonatal nurse practitioner	216	(52)
HCA (healthcare assistant)	196	(47)
Consultant anaesthetist with regular sessions in obstetrics	179	(43)
MSW (maternity support worker)	172	(41)
Consultant obstetrician	157	(38)
Trainee anaesthetist not on obstetric rotation	57	(14)
Consultant neonatologist	32	(8)
Consultant anaesthetist without regular sessions in obstetrics	24	(6)
Midwife band 8-9	18	(4)
Other	38	(9)

SAS doctor = staff grade, associate specialist and specialty doctor.

Table S3. Acceptable and unacceptable techniques and adjunctive measures to manage IFH at caesarean birth as indicated by consultant obstetricians (n=145), trainee/registrars/specialty obstetricians (n=71), and midwives (144).

Techniques and adjunctive measures	Acceptable		Unacceptable		I don't know	
	n	(%)	n	(%)	n	(%)
**Change of operator (different clinician)**						
Consultant obstetricians	139	(96)	2	(1)	4	(3)
Trainee/registrars/trust doctor obstetricians	68	(96)	1	(1)	2	(3)
Midwives	119	(83)	5	(4)	20	(14)
**Manual cephalic extraction using usual delivering hand**						
Consultant obstetricians	139	(96)	6	(4)	0	(0)
Trainee/registrars/trust doctor obstetricians	69	(97)	1	(1)	1	(1)
Midwives	89	(62)	8	(6)	47	(33)
**Tocolysis (GTN / Terbutaline / Salbulatmol)**						
Consultant obstetricians	129	(89)	15	(10)	1	(1)
Trainee/registrars/trust doctor obstetricians	67	(94)	1	(1)	3	(4)
Midwives	83	(58)	9	(6)	52	(36)
**Operator changing hand to perform manual cephalic extraction**						
Consultant obstetricians	127	(88)	10	(7)	8	(6)
Trainee/registrars/trust doctor obstetricians	70	(99)	1	(1)	0	(0)
Midwives	72	(50)	10	(7)	62	(43)
**Reverse breech extraction**						
Consultant obstetricians	122	(84)	9	(6)	14	(10)
Trainee/registrars/trust doctor obstetricians	70	(99)	1	(1)	0	(0)

Midwives	75	(52)	15	(10)	54	(38)
**Fetal pillow® (fetal head elevating device)**						
Consultant obstetricians	118	(81)	4	(3)	23	(16)
Trainee/registrars/trust doctor obstetricians	54	(76)	3	(4)	14	(20)
Midwives	99	(69)	2	(1)	43	(30)
**Head down tilt**						
Consultant obstetricians	115	(79)	13	(9)	17	(12)
Trainee/registrars/trust doctor obstetricians	67	(94)	1	(1)	3	(4)
Midwives	66	(46)	15	(10)	63	(44)
**Vaginal push-up (after incision)**						
Consultant obstetricians	109	(75)	29	(20)	7	(5)
Trainee/registrars/trust doctor obstetricians	55	(78)	12	(17)	4	(6)
Midwives	66	(46)	15	(10)	63	(44)
**Vaginal push-up (pre-incision)**						
Consultant obstetricians	101	(70)	33	(23)	11	(8)
Trainee/registrars/trust doctor obstetricians	55	(78)	8	(11)	8	(11)
Midwives	36	(25)	64	(44)	44	(31)
**Patwardhan method (shoulders first)**						
Consultant obstetricians	61	(42)	22	(15)	62	(43)
Trainee/registrars/trust doctor obstetricians	29	(41)	9	(13)	33	(47)
Midwives	18	(13)	19	(13)	107	(74)
**Bladder filling**						
Consultant obstetricians	19	(13)	67	(46)	59	(41)
Trainee/registrars/trust doctor obstetricians	13	(18)	20	(28)	38	(54)
Midwives	23	(16)	42	(29)	79	(55)

**Single forceps blade**						
Consultant obstetricians	21	(15)	92	(64)	32	(22)
Trainee/registrars/trust doctor obstetricians	13	(18)	30	(42)	28	(39)
Midwives	18	(13)	61	(42)	65	(45)
**Ventouse**						
Consultant obstetricians	15	(10)	97	(67)	33	(23)
Trainee/registrars/trust doctor obstetricians	7	(10)	40	(56)	24	(34)
Midwives	5	(4)	93	(65)	46	(32)
**Tydeman tube**						
Consultant obstetricians	14	(10)	5	(4)	126	(87)
Trainee/registrars/trust doctor obstetricians	2	(3)	2	(3)	67	(94)
Midwives	7	(5)	1	(1)	136	(94)
**C-snorkel**						
Consultant obstetricians	8	(6)	4	(3)	133	(92)
Trainee/registrars/trust doctor obstetricians	2	(3)	1	(1)	68	(96)
Midwives	3	(3)	1	(1)	139	(97)

GTN = Glyceryl trinitrate

Table S4. Vaginal disimpaction ("push-up") in current practice of midwives (n=144) and obstetricians (n=216)

**How vaginal "push-up" is performed in current practice** (participants could select all that apply)	**Percentage (count) of midwives (n=144)**		**Percentage (count) of obstetricians (n=216)**	
	**n**	**(%)**	**n**	**(%)**
Using a whole hand to disimpact the fetal head	89	(62)	172	(80)
Using the same technique as for standard vaginal examination	32	(22)	26	(12)
Flex and abduct both of the woman's legs after an IFH is diagnosed and when the decision is made to perform a vaginal push up	62	(43)	97	(45)
Flex and abduct one of the woman's legs to achieve access to perform the vaginal push up	17	(12)	48	(22)
Perform the manoeuvre without re-positioning the woman's legs from a supine position	39	(27)	31	(14)
Flex and abduct both of the woman's legs prior to commencing the caesarean section, if there is an increased risk of IFH	6	(4)	44	(20)
None of the above	12	(8)	12	(6)
Other	17	(12)	30	(14)

IFH = impacted fetal head
